# Transmission of SARS-CoV-2 in children aged 0 to 19 years in childcare facilities and schools after their reopening in May 2020, Baden-Württemberg, Germany

**DOI:** 10.2807/1560-7917.ES.2020.25.36.2001587

**Published:** 2020-09-10

**Authors:** J Ehrhardt, A Ekinci, H Krehl, M Meincke, I Finci, J Klein, B Geisel, C Wagner-Wiening, M Eichner, SO Brockmann

**Affiliations:** 1 Department of Health Protection and Epidemiology, Baden-Wuerttemberg State Health Office, Stuttgart, Germany; 2 These authors contributed equally; 3 Project Containment Scouts, Federal Office of Administration, Cologne, Germany; 4 Postgraduate Training for Applied Epidemiology (PAE), Robert Koch-Institute Berlin, Germany; 5 European Programme of Intervention Epidemiology Training (EPIET), Stockholm, Sweden; 6 Department of Hygiene and Infection Control, Baden-Wuerttemberg State Health Office, Stuttgart, Germany; 7 Institute for Clinical Epidemiology and Applied Biometry, University of Tübingen, Germany

**Keywords:** school, transmission, SARS-CoV-2, COVID-19

## Abstract

We investigated data from severe acute respiratory syndrome coronavirus 2 (SARS-CoV-2) infected 0–19 year olds, who attended schools/childcare facilities, to assess their role in SARS-CoV-2 transmission after these establishments’ reopening in May 2020 in Baden-Württemberg, Germany. Child-to-child transmission in schools/childcare facilities appeared very uncommon. We anticipate that, with face mask use and frequent ventilation of rooms, transmission rates in schools/childcare facilities would remain low in the next term, even if classes’ group sizes were increased.

To gain further understanding on paediatric transmission of severe acute respiratory syndrome coronavirus 2 (SARS-CoV-2) in the school/childcare-facility context, we compiled and analysed data from SARS-CoV-2 infected children (age: 0–19 years), who had been to school/childcare facilities, after such establishments reopened in Baden-Württemberg in May 2020.

## Reopening of schools/childcare facilities in Baden-Württemberg

Closure of schools and childcare facilities was part of the German national response and containment strategy of SARS-CoV-2, like in most other European Union countries [1]. In the federal state of Baden-Württemberg in south-west Germany, which has a population of 10.8 million, school and childcare facility closures were mandated on 17 March 2020. From that time, some emergency childcare facilities were nevertheless established for children whose parents both worked in essential services. On 27 April, they were extended to children of persons who could not work from home; for all others, childcare facilities finally reopened on 29 June. Concerning schools, almost 2 months after closing, these reopened in a stepwise manner, beginning on 4 May with the graduating classes of secondary schools, followed on 18 May by the graduating classes of primary schools, and finally, on 15 June, by all remaining classes. The reopening of schools and childcare facilities was accompanied by a series of measures to prevent the spread of SARS-CoV-2 (Table 1). 

**Table 1:** Infection control measures for the prevention of SARS-CoV-2 transmission in schools and childcare facilities in Baden-Württemberg, Germany, May–July 2020

Infection control measure	Childcare facilities	Primary school	Secondary school^a^
Group sizes reduced by 50%	Yes	Yes	Yes
Cleaning of contact surfaces	Yes	Yes	Yes
Regular and interim ventilation of rooms	Yes	Yes	Yes
Exclusion of sick children	Yes	Yes	Yes
Individual hygiene (hand hygiene, cough etiquette)	Yes	Yes	Yes
Face mask in classroom	No	No	No
Face mask outside classroom	No	Some	Some
Physical distancing between children	No	No	Yes
Cancelling singing and use of wind instruments during music lesson	Some	Yes	Yes
Cancelling physical education	NA	Yes	Yes

NA: not applicable; SARS-CoV-2: severe acute respiratory syndrome coronavirus 2.

^a ^Including vocational school.

White backgrounds indicate measures which will remain unchanged after reopening following the 2020 summer holidays; light blue backgrounds indicate measures that will be cancelled after the 2020 summer holidays; dark blue backgrounds indicate measures that will be established (mask outside classroom) or should be established in the authors’ opinion (ventilation and mask in classrooms).

## Data source, study period and epidemiological investigation

**Figure 1:**
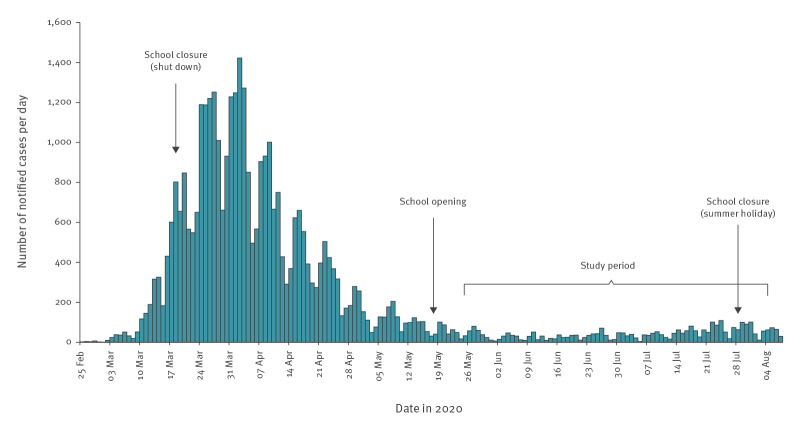
Daily number of notified COVID-19 cases in Baden-Württemberg, by date of reporting, Germany, 25 February–07 August 2020 (n = 37,752)

To assess the viral transmission role of SARS-CoV-2-infected children who attended schools and childcare facilities after their reopening, we searched all notified (i.e. laboratory-confirmed) coronavirus disease (COVID-19) cases from the state of Baden-Württemberg. Data on all cases aged 0–19 years in the period from 25 May to 5 August 2020 (i.e. from 1 week after school opening in May until 1 week after school closure due to the summer holidays; Figure 1) were compiled.

COVID-19: coronavirus disease.

Arrows show the dates of school closing and opening. The bracket indicates the study period, from 1 week after schools had completely reopened (25 May 2020) to 1 week after the beginning of summer holiday (5 August 2020).

We contacted the notifying local health offices and reinvestigated school-attendance during the presumed infectious period of these cases, which was according the national standards of the Robert Koch Institute assumed to start 2 days before the onset of symptoms or, in case of an asymptomatic infection, 48 hours before the sampling date of the positive test result [2]. Upon identifying cases, the local health offices had initiated thorough contact investigations in the schools and childcare facilities respectively.

## Ethical statement

This analysis was conducted as part of public health usual practice, and was not conducted for research. Ethics approval was, therefore, not needed.

## Cases and transmission events in schools and childcare settings

**Figure 2:**
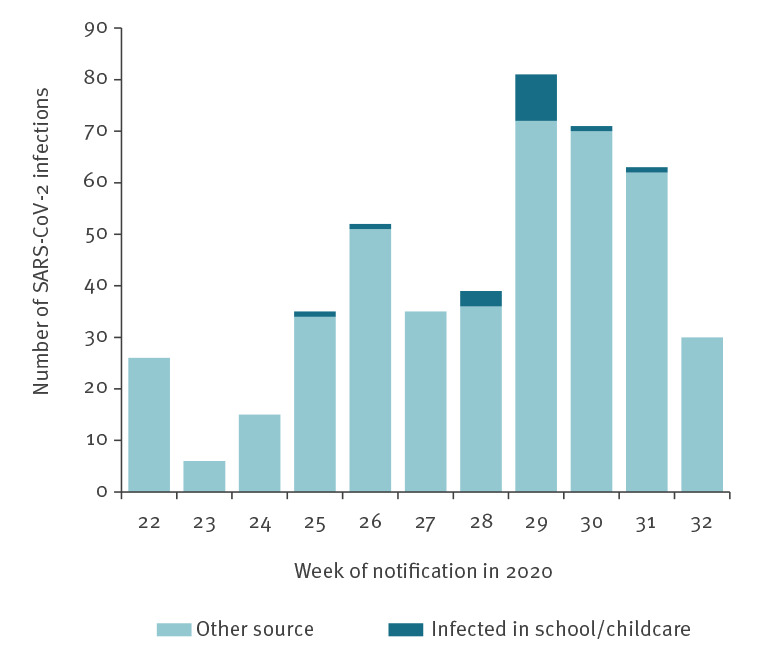
Weekly number of notified SARS-CoV-2 infections in the age group 0–19 years by source of infection, Baden-Württemberg, Germany, 25 May–2 August 2020 (n = 453)

In total, 557 cases of age 0–19 years were notified during the study period in Baden-Württemberg (17.9% of all 3,104 notified cases) and for 453 (81.3%) information on school attendance was available; 137 (30%) of these 453 cases attended school or childcare settings for at least 1 day in their infectious period whereas the remaining 316 were at home during their entire infectious period. More than 2,300 nasopharyngeal swabs were taken from the close contacts (teachers and pupils) of the 137 index cases, and from the close contacts of any secondary cases, if identified. Swabbing usually occurred 3 to 5 days after the index cases’ diagnosis. Six of the 137 cases were found to have infected a total of 11 additional pupils (one to three pupils per case; see Figure 2; three in childcare facilities, one in primary school, four in secondary school and three in vocational school), whereas no secondary infections could be detected for the remaining cases despite extensive contact tracing and swabbing of school and childcare-facility contacts. To the best of our knowledge, aside from the 11 secondary cases and another four pupils who were infected by two teachers, all remaining cases with information on school attendance (n = 437) were caused by sources outside of school and childcare facilities (Table 2). 

SARS-CoV-2: severe acute respiratory syndrome coronavirus 2.

Cases infected in school/childcare are shown in dark blue, cases infected by other sources in light blue.

**Table 2:** Source of SARS-CoV-2 infection in persons aged 0–19 years, Baden-Württemberg, Germany, 25 May–5 August 2020 (n = 453)^a^

Setting/source of infection	Number of infected persons	Percentage
**Household**	**190**	**41.9%**
Parents	93	NA
Grandparents	13	NA
Siblings	7^b^	NA
Not specified	77	NA
**Festivity/event^c^**	**38**	**8.4%**
**School/childcare**	**15**	**3.3%**
By pupil	11	NA
By teacher	4	NA
**Church/community of faith**	**14**	**3.1%**
**Travel associated**	**5**	**1.1%**
**Others**	**4**	**0.9%**
**Unknown or not available^d^**	**187**	**41.3%**

NA: not applicable; SARS-CoV-2: severe acute respiratory syndrome coronavirus 2.

**^a ^**Of the 557 children aged 0–19 years, who were notified with SARS-CoV-2 infection, data on school attendance were available for 453. Information on these 453 children is presented in the table.

**^b^** Seven children infected in three intra-household clusters.

**^c ^**Birthdays and other parties, weddings, funerals.

^d^ As close contacts of the cases were thoroughly examined, it is unlikely that cases in the ‘unknown’ category were infected in childcare facilities, schools or private households.

Assuming that every one of the 137 index cases spent on average 2 days at school during the infectious period, the 11 secondary cases originated from a cumulative number of 274 infectious days, i.e. one secondary case per roughly 25 infectious school days.

## Discussion and conclusion

There is an ongoing discussion in the scientific community regarding the role of children in the transmission of SARS-CoV-2. Recently, the percentage of children and adolescents up to 19 years old among all COVID-19 cases in Germany has increased to 25% [3]. Infected children are more likely to remain asymptomatic or have a mild course of disease and are much less likely than adults to be hospitalised or have fatal outcomes. Thus, their infection may go undetected or undiagnosed. Symptomatic children seem to shed virus in similar quantities as adults and can infect others in a similar way, but it is unknown how infectious asymptomatic children are [1,4,5].

Our investigation suggests that child-to-child transmission in schools and childcare facilities is uncommon and not the primary cause of SARS-CoV-2 infection in children. Based on our estimation there could be one secondary case per roughly 25 infectious school days. This ratio of 1 in 25 might, however, overestimate the transmission risk in schools and childcare facilities, because some of the 104 index cases (i.e. 104 = 557 − 453) for whom no information on school attendance was available, may also have spent some time in school or in a childcare facility while being infectious, yet without further generating any notified COVID-19 cases. While investigations from Ireland concur with our results [6], a report from Israel showed a large outbreak in apparently over-crowded schools where face-mask usage had been discontinued due to a heat wave [7].

The low transmission in schools and childcare facilities found in this current study might be due in part to the infection control measures initiated after school/childcare-facility reopening, yet it is not clear how much the different measures have contributed. In order to gradually return to the regular school and childcare-facility life, larger classes will have to be accepted again. This will require more proximity between pupils. As a countermeasure, strict ventilation of classrooms, not only between lessons but also within, should be implemented [1]. Additionally, face masks should be used in schools, both, inside and outside of classrooms. Based on our current study findings, we anticipate that transmission rates in schools and childcare facilities would remain low under such interventions [8].
